# Understanding Integrated Care

**DOI:** 10.5334/ijic.2530

**Published:** 2016-10-28

**Authors:** Nick Goodwin

**Affiliations:** International Foundation for Integrated Care, IJIC, 10 Goldsmith Close, Bicester, GB

## Introduction

Integrated care is a concept that is now commonly accepted across the world yet there remains a persistent and enduring ‘confusion of languages’ when it comes to understanding it [1]. This perspective paper seeks to bring a degree of clarity to the meaning of integrated care. It argues that integrated care cannot be narrowly defined, but should be seen as an overarching term for a broad and multi-component set of ideas and principles that seek to better co-ordinate care around people’s needs.

## How is integrated care defined?

It is well known that integrated care has been provided with many different definitions [2]. This diversity has been driven by the different purposes (all legitimate) that various stakeholders within care systems attribute to the term [3]. For example, this can be driven by differing professional points of view (e.g. clinical vs. managerial; professional vs. patient) or from the disciplinary perspective of the observer (e.g. public administration, public health, social science, or psychology) [4].

Some of the most commonly used definitions from these different perspectives can be found in Box 1 [5,6,7,8]. These demonstrate two principle characteristics of integrated care as a concept. First, it must involve bringing together key aspects in the design and delivery of care systems that are fragmented (i.e. ‘to integrate’ so that parts are combined to form a whole). Second, that the concept must deliver ‘care’, which in this context would refer to providing attentive assistance or treatment to people in need. Integrated care, then, results when the former (integration) is required to optimise that latter (care).

**Box 1: Four Commonly Used Definitions of Integrated Care**

**A health system-based definition**

“Integrated health services: health services that are managed and delivered so that people receive a continuum of health promotion, disease prevention, diagnosis, treatment, disease-management, rehabilitation and palliative care services, coordinated across the different levels and sites of care within and beyond the health sector, and according to their needs throughout the life course.” [4]

**A managers’ definition**

“The process that involves creating and maintaining, over time, a common structure between independent stakeholders … for the purpose of coordinating their interdependence in order to enable them to work together on a collective project” [5]

**A social science-based definition**

“Integration is a coherent set of methods and models on the funding, administrative, organizational, service delivery and clinical levels designed to create connectivity, alignment and collaboration within and between the cure and care sectors. The goal of these methods and models is to enhance quality of care and quality of life, consumer satisfaction and system efficiency for people by cutting across multiple services, providers and settings. Where the result of such multi-pronged efforts to promote integration lead to benefits for people the outcome can be called ‘integrated care’” [adapted from 6]

**A definition based on the perspective of the patient (person-centred coordinated care)**

“I can plan my care with people who work together to understand me and my carer(s), allow me control, and bring together services to achieve the outcomes important to me.” [7]

Despite the basic simplicity this understanding presents, it is a truism to say that the experience of those implementing integrated care programmes take a significant amount of time to define and interpret what it will mean to them in their own contexts. This is important as none of the standard definitions quite work in all circumstances, so it important that partners in care agree upon the details of their own version rather than pick one of the shelf. Nonetheless, it is important to avoid the tendency to focus on structural or organisationally-based definitions, or those that focus purely on integration as a means to create cost efficiencies. Rather, by providing a ‘people-centred’ definition with the core purpose of ‘caring’ so integrated care is given a compelling logic as to its objectives and how success might be judged [8].

## What forms does integrated care take?

Integrated care is characterised by complexity. However, a number of different conceptual frameworks and taxonomies have been developed to help manage our understanding, Typically, these have examined [2,9]:

- the *type* of integration (i.e. organisational, professional, cultural, technological);
- the *level* at which integration occurs (i.e. macro-, meso- and micro-);
- the *process* of integration (i.e. how integrated care delivery is organised and managed);
- the *breadth* of integration (i.e. to a whole population group or specific client group); and
- the *degree* or *intensity* of integration (i.e. across a continuum that spans between informal linkages to more managed care co-ordination and fully integrated teams or organisations).

Moreover, integrated care takes a number of key forms, including [10]:

- *Horizontal integration.* Integrated care between health services, social services and other care providers that is usually based on the development of multi-disciplinary teams and/or care networks that support a specific client group (e.g. for older people with complex needs)
- *Vertical integration.* Integrated care across primary, community, hospital and tertiary care services manifest in protocol-driven (best practice) care pathways for people with specific diseases (such as COPD and diabetes) and/or care transitions between hospitals to intermediate and community-based care providers
- *Sectoral integration.* Integrated care within one sector, for example combining horizontal and vertical programmes of integrated care within mental health services through multi-professional teams and networks of primary, community and secondary care providers;
- *People-centred integration:* Integrated care between providers and patients and other service users to engage and empower people through health education, shared decision-making, supported self-management, and community engagement; and
- *Whole-system integration:* Integrated care that embraces public health to support both a population-based and person-centred approach to care. This is integrated care at its most ambitious since it focuses on the multiple needs of whole populations, not just to care groups or diseases.

It is often suggested that the strongest form is the ‘fully integrated’ model that is characterised by integrated teams working in an organisation with a single set of governance and accountability rules and common budgets and incentives [1]. Indeed, there is evidence to suggest that the more severe the need of the patient, the more appropriate it might be to develop ‘fully integrated’ organisations [11]. Yet, what appears to matter most is not the organisational solution but what happens at the service- and clinical-level [12]. Transformational change can only happen at the interface between service users and teams of care professionals working in partnership with them. For people with complex needs, this implies a more flexible and networked solution where a ‘core team’ empowers service users and supports their day-to-day needs but can rely on a responsive provider network when required [13].

## How has our understanding of integrated care developed?

Our understanding of what integrated care means, and what it might comprise, continues to evolve. In many respects, we now know the basic building blocks of a successful integrated care approach since there have been numerous studies developing frameworks through which the different elements are set out [e.g. see 14,15,16,17]. One of the unmet challenges is how we might move beyond these descriptive components to offer a guide to decision-makers on how best to implement integrated care in policy and practice. In this respect, we know that much depends on the ‘softer issues’ of relationship building and the ability to foster an environment where new collaborations and ways of working become accepted as the norm over time.

In more recent years, too, a number of new ideas have emerged that have taken our understanding of integrated care along a different path. The two most fundamental of these include: first, the recognition that engaging and empowering people and communities should be a central component to any integrated care strategy; and second, that integrated care strategies might be most powerful where they become population-oriented and focused on promoting health, for example by bringing together health and social care with other players such as housing, schools, community groups, industry, and so on. Both these ideas see the integration element as a way of bringing community assets together to promote health and wellbeing to populations, so taking the potential focus of integrated care beyond specific service models or the propensity to individualise the focus around, for example, disease management programmes and care pathways. A debate is then created as to whether integrated care should be underpinned by a set of core ‘values’, such as equity or solidarity, which brings us full circle into the debate about integrated care’s meaning [18].

## Conclusions

At its simplest, integrated care is an approach to overcome care fragmentations, especially where this is leading to an adverse impact on people’s care experiences and care outcomes. Integrated care may be best suited to people with medically complex or long-term care needs, yet the term should not be solely regarded as a means to managing medical problems since the principles extend to the wider definition of promoting health and wellbeing. Indeed, it seems that whilst our understanding of integrated care has advanced it also continues to evolve and be debated. At its heart, however, lies a commitment to improving the quality and safety of care services through ongoing and co-productive partnerships.

## References

[1] Kodner D (2009). All together now: a conceptual exploration of integrated care. Healthcare Quarterly.

[2] Armitage GD, Suter E, Oelke ND, Adair CE (2009). Health systems integration: state of the evidence. International Journal of Integrated Care.

[3] World Health Organisation (2016). Framework on integrated, people-centred health services. Report by the Secretariat. Sixty-Ninth World Health Assembly.

[4] Contandriapoulos AP, Denis JL, Touati N, Rodriguez C (2003). Groupe de recherche interdisciplinaire en santé. Working Paper N04–01. http://nelhin.on.ca/assets/0/16/2100/3734/3736/6cab135d-87c1–45bd-88cd-2c1d5404ec9b.pdf.

[5] Kodner D, Spreeuwenberg C (2002). Integrated care: meaning, logic, applications and implications – a discussion paper. International Journal of Integrated Care.

[6] National Voices (2013). A narrative for person-centred coordinated care. http://www.nationalvoices.org.uk/sites/default/files/public/publications/narrative-for-person-centred-coordinated-care.pdf.

[7] Lewis R, Rosen R, Goodwin N, Dixon J (2010). Where next for integrated care organisations in the English NHS?.

[8] Goodwin N, Alonso A, Muller S, Meyer I, Kubitschke L (2014). Understanding integrated care: the role of information and communication technology. Beyond Silos: The way and how of eCare.

[9] Goodwin N, Smith J (2012). The evidence base for integrated care. http://www.nuffieldtrust.org.uk/sites/files/nuffield/evidence-base-for-integrated-care-251011.pdf.

[10] Leutz WN (1999). ‘Five laws for integrating medical and social services: lessons from the United States and the United Kingdom’. Milbank Quarterly.

[11] Curry N, Ham C (2010). Clinical and Service Integration: The route to improved outcomes.

[12] Goodwin N, Sonola L, Thiel V, Kodner D (2013). Co-ordinated care for people with complex chronic conditions. Key lessons and markers for success.

[13] Fulop N, Mowlam A, Edwards N (2005). Building integrated care. Lessons from the NHS and elsewhere.

[14] Minkman M, Ahaus KT, Huijsman R (2009). A four phase development model for integrated care services in the Netherlands. BMC Health Service Research.

[15] Valentijn PP, Schepman SM, Opheij W, Bruijnzeels M A (2013). ”Understanding integrated care: a comprehensive conceptual framework based on the integrative functions of primary care”. Int.J.Integr.Care.

[16] WHO Regional Office for Europe (2013). Strengthening people-centred health systems in the WHO European Region: a roadmap.

[17] Calciolari S, Gonzalez L, Goodwin N, Stein V (2016). International Check: Conceptual framework and comparative assessment exercise.

[18] Minkman MMN (2016). Values and Principles of Integrated Care. International Journal of Integrated Care.

